# New Insights into Mechanisms of Action for Omega-3 Fatty Acids in Atherothrombotic Cardiovascular Disease

**DOI:** 10.1007/s11883-019-0762-1

**Published:** 2019-01-12

**Authors:** R. Preston Mason

**Affiliations:** 1 Cardiovascular Division, Department of Medicine, Brigham and Women’s Hospital, Harvard Medical School, Boston, MA 02115 USA; 2 Elucida Research LLC, Beverly, MA 01915 USA

**Keywords:** Atherosclerosis, Triglycerides, Lipoproteins, Omega-3 fatty acids, Cholesterol, Eicosapentaenoic

## Abstract

**Purpose of Review:**

Treatment of hypercholesterolemia with statins results in significant reductions in cardiovascular risk; however, individuals with well-controlled low-density lipoprotein cholesterol (LDL-C) levels, but persistent high triglycerides (TG), remain at increased risk. Genetic and epidemiologic studies have shown that elevated fasting TG levels are associated with incident cardiovascular events. At effective doses, omega-3 fatty acids, such as eicosapentaenoic acid (EPA) and docosahexaenoic acid (DHA), lower TG levels but may have additional atheroprotective properties compared to other TG-lowering therapies such as niacin and fibrates. The purpose of this review is to evaluate mechanisms related to the potential benefits of omega-3 fatty acids in atherothrombotic disease.

**Recent Findings:**

Large randomized clinical trials are currently under way to test the cardiovascular benefits of omega-3 fatty acids at a pharmacologic dosage (4 g/day). A large randomized trial with a prescription EPA-only formulation was shown to reduce a composite of cardiovascular events by 25% in statin-treated patients with established cardiovascular disease or diabetes and other CV risk factors. EPA and DHA have distinct tissue distributions as well as disparate effects on membrane structure and lipid dynamics, rates of lipid oxidation, and signal transduction pathways. Compared to other TG-lowering therapies, EPA has been found to inhibit cholesterol crystal formation, inflammation, and oxidative modification of atherogenic lipoprotein particles. The anti-inflammatory and endothelial benefits of EPA are enhanced in combination with a statin.

**Summary:**

Omega-3 fatty acids like EPA only at a pharmacologic dose reduce fasting TG and interfere with mechanisms of atherosclerosis that results in reduced cardiovascular events. Additional mechanistic trials will provide further insights into their role in reducing cardiovascular risk in subjects with well-managed LDL-C but elevated TG levels.

## Introduction

While statins have significantly reduced the global burden of atherosclerotic cardiovascular disease (ASCVD), there is still residual risk for clinical events among patients, such as those with type 2 diabetes mellitus (DM) or other metabolic diseases [1]. This has led to research into additional lipid targets for intervention, including high-density lipoprotein cholesterol (HDL-C) and triglycerides (TG). While treatments directed towards HDL-C have been disappointing, there is epidemiologic and genetic data that suggest elevated TG levels are an important risk factor for cardiovascular disease [2•]. However, it remains a challenge to determine the extent to which TGs are an independent risk factor after adjusting for other lipid parameters [3, 4]. In Mendelian genetic studies, causal relationships are also difficult to confirm as variants may have pleiotropic effects that serve as confounders with respect to disease progression [5•]. Clinical trials that have tested TG-lowering agents have failed to show significant cardiovascular benefits when added to statins as compared to statin therapy alone. An important limitation of these trials, such as those with niacin or fenofibrate [6–9], is that patients with elevated TG levels were not enrolled prospectively. However, a recent trial with a prescription EPA-only formulation was shown to reduce a composite of cardiovascular events by 25% in statin-treated patients with elevated and high baseline fasting TG levels (135–499 mg/dL) along with elevated cardiovascular risk [10••]. This trial is supported by emerging lines of evidence that O3FAs like EPA reduce inflammation, LDL-C oxidation, and endothelial dysfunction. The results of this and other outcome studies should provide important insights as to how O3FAs may influence the etiology of ASCVD.

## Omega-3 Fatty Acids and Atherothrombotic Disease

Consumption of marine-derived, long-chain, polyunsaturated O3FAs has been shown to significantly reduce TG and there is suggestive data that report reduced risk for cardiovascular mortality and morbidity [11, 12, 13••, 14, 15, 16•, 17•]. However, recent ASCVD outcome trials have not shown any CV benefit with low doses (1 g/day) of omega-3 mixtures containing EPA and DHA on top of contemporary medical therapy [18•, 19]. In the Japan EPA Lipid Intervention Study (JELIS) trial, purified EPA (1.8 g/day) was effective in reducing the risk of major coronary events in hypercholesterolemic patients receiving statin therapy versus those subjected to statin monotherapy [11]. Also, coronary plaque regression was observed in statin-treated patients in the Combination Therapy of Eicosapentaenoic Acid and Pitavastatin for Coronary Plaque Regression Evaluated by Integrated Backscatter Intravascular Ultrasonography (CHERRY) study using 1.8 g/day EPA only [17•]. Other studies are difficult to interpret as they used heterogeneous O3FA formulations with respect to dosing levels and purity [16•]. In particular, many of these trials utilized low doses of O3FAs with varying ratios of EPA and DHA or even unregulated dietary fish oil supplements [16•].

Although EPA and DHA have similar effects on TG levels, there is growing evidence these O3FA may differentially affect rates of lipid oxidation, membrane structure, and various cellular functions [13••, 14, 15]. In clinical studies of patients with high (200–500 mg/dL) or very high TG levels (≥ 500 mg/dL), prescription EPA-only treatment has been reported to reduce high-sensitivity C-reactive protein (hsCRP), lipoprotein-associated phospholipase A_2_ (Lp-PLA_2_), and oxidized LDL-C (oxLDL) levels, as well as the arachidonic acid (AA)-to-EPA ratio, as compared to placebo [19–25]. In comparison to DHA, EPA administration was associated with significant reductions in the expression of genes linked to cardiovascular disease, including those involved in the interferon pathway [26]. EPA treatment also resulted in the downregulation of the cAMP responsive element protein 1 (CREB1) and hypoxia inducible factor 1 (HIF1) gene expression [26]. In cellular studies, EPA reduced production of inflammatory mediators such as TNF-α and IL-1β compared to DHA in alveolar macrophages following LPS stimulation [27]. There is one randomized clinical trial currently under way that is adequately powered to evaluate the cardiovascular benefits of combined statin and high-dose prescription O3FA therapy in patients with elevated TG levels [2•]. There are also two low to moderate dose O3FA (1–2 g/day) trials using varying ratios of EPA and DHA to assess the CV benefit in a heterogenous patient population with regard to CV risk. These are reviewed in Table 1.

**Table 1:** Ongoing randomized controlled trials with omega-3 fatty acids and cardiovascular disease

Trial (location)	*N*	Age (years)	Design	Formulation, dose	Duration (years)	Expected completion date	Inclusion criteria or cohort characteristics
STRENGTH (USA)	13,086	18–99 (> 40 if diabetes)	Secondary prevention of CVD; primary if diabetes with risk factors	EPA + DHA carboxylic acids, 4 g	5	2020	LDL-C < 100 mg/dL, on statin; TG 180–499 mg/dL; HDL-C < 42 mg/dL in men, < 47 mg/dL in women; patients with CVD or diabetes with risk factors
RESPECT-EPA (Japan)	3900	20–79	Stable CAD open-label	EPA, 1.8 g	5	2022	Statin treated; patients with stable CAD
OMEMI (Norway)	1400	70–82	Secondary prevention	EPA + DHA, 1.8 g	2–4	2020	Statin-treated patients with post-MI, stable

*CAD* coronary artery disease, *CVD* cardiovascular disease, *DHA* docosahexaenoic acid, *EPA* eicosapentaenoic acid, *HDL-C* high-density lipoprotein cholesterol, *LDL-C* low-density lipoprotein cholesterol, *MI* myocardial infarction, *OMEMI* Omega-3 Fatty Acids in Elderly Patients with Myocardial Infarction, *REDUCE-IT* Reduction of Cardiovascular Events with Icosapent Ethyl-Intervention Trial, *RESPECT-EPA* Randomized Trial for Evaluation in Secondary Prevention Efficacy and Combination Therapy-Statin and EPA, *STRENGTH* Statin Residual Risk Reduction with EpaNova in High Cardiovascular Risk, *TG* triglyceride(s), *VITAL* Vitamin D and Omega A3 trial

The Reduction of Cardiovascular Events with Icosapent Ethyl–Intervention (REDUCE-IT; NCT01492364) trial was a randomized, double-blinded, placebo-controlled trial designed to examine the benefits of icosapent ethyl, a prescription, highly purified ethyl ester of EPA [28]. The primary objective of this study was to determine if robust EPA treatment reduces ischemic events in statin-treated patients with elevated and high baseline fasting TG levels (150–499 mg/dL) and elevated cardiovascular risk for clinical events. The trial enrolled 8179 men and women at or above 45 years of age with established ASCVD or above 50 years of age with type 2 diabetes and one additional risk factor. Randomization required stable statin (± ezetimibe) treatment for at least 4 weeks prior to qualifying measurements and fasting TG and LDL-C levels of 150–499 and 41–100 mg/dL, respectively. The primary endpoint was a composite of time to first event for cardiovascular death, nonfatal MI, nonfatal stroke, coronary revascularization, or hospitalization for unstable angina. The primary composite endpoint reported a highly statistically significant (*p* < 0.001) 25% relative risk reduction (RRR) and 4.8% absolute risk reduction (ARR) with a number needed to treat (NNT) of 21 over 4.9 years [10••]. The secondary composite endpoint (three-point MACE of CV death, nonfatal MI, nonfatal stroke) also demonstrated a highly statistically significant (*p* < 0.001) 26% RRR (3.6% ARR) an NNT of 28 over 4.9 years. Reductions in other secondary endpoints were also significant, including a 20% reduction in CV death, 31% reduction in MI, and a 28% reduction in stroke. Tertiary endpoints of sudden cardiac death and cardiac arrest were also reduced by 31% (HR[95% CI], 0.69 [0.50–0.96]) and 48% (HR[95% CI], 0.42 [0.31–0.86]). While overall adverse event rates were similar across treatment groups, there were numerically more serious adverse events related to bleeding; overall rates were low (2.7% for EPA vs 2.1% for placebo, *p* = 0.06), with no fatal bleeding observed in either group and no significant increase in adjudicated hemorrhagic stroke or serious central nervous system or gastrointestinal bleeding. There was a significantly higher rate of hospitalization for atrial fibrillation or flutter, though rates were low (3.1% for EPA vs 2.1% for placebo, *p* = 0.004). The rate of atrial fibrillation did not appear to be clinically meaningful as the treatment was associated with significantly lower rates of stroke (28% relative risk reduction in fatal or nonfatal stroke, *p* < 0.01).

The Statin Residual Risk Reduction with EpaNova in High Cardiovascular Risk (STRENGTH) trial is expected to be reported in 2020 [29]. It is also a randomized, double-blinded, placebo-controlled study but differs from REDUCE-IT in that high-risk patients, with elevated TG levels, are treated with 4 g/day of free fatty acid formulations of EPA and DHA. STRENGTH imposes an additional requirement of an HDL-C level less than 40 mg/dL at baseline. The OMEMI and RESPECT-EPA trials all are using lower doses of O3FA (≤ 2 g/day) of mixed EPA and DHA or pure EPA in patients with different categories of cardiovascular risk and limited statin use (Table 1).

Recently, the ASCEND trial reported the effect of O3FA supplementation in 15,480 patients with diabetes but without evidence of atherosclerotic cardiovascular disease [18•]. The study was a 2 × 2 factorial design, randomized study to assess whether aspirin 100 mg/day versus placebo and separately, mixed O3FA 1 g/day versus placebo, reduce the risk of serious vascular events. Statin use was not required for this trial. The results failed to demonstrate a reduction of first serious vascular events in the O3FA arm. This is consistent with most prior studies of O3FA mixtures at low doses [2•, 16•]. The failure of the ASCEND study to demonstrate cardiovascular benefit is further motivation to test higher dose O3FA, including EPA only, in patients with atherosclerotic disease. A similar failure was reported in the VITAL study among more than 25,000 patients with low CV risk using again a low-dose O3FA 1 g/day [30•]. In this primary prevention trial, there was no statistically significant decrease in the primary composite cardiovascular endpoint or cancer-associated endpoints. While there was a reduction in certain secondary endpoints such MI, these are only hypothesis generating and require an appropriately powered study to test. Thus, both the ASCEND and VITAL trials showed a consistent lack of benefit in over 40,000 patients using a low-dose O3FA mixture with respect to primary prevention.

## Fish Oil Supplements as a Source of O3FAs for Cardiovascular Disease

There is considerable debate about fish oil dietary supplements (FODS) and whether or not they contain levels of quality O3FAs adequate for treating patients with cardiovascular risk and/or elevated TGs. According to a 2008 report by the United States Department of Health and Human Services on complementary and alternative medicine use among adults and children, FODS are the most commonly used supplements among adults in the USA [31]. There is a general public perception that consumption of fish is a healthy dietary habit [32]. Indeed, fish consumption is recommended in the 2015–2020 Dietary Guidelines for Americans and by the American Heart Association [33••]. This view has led to a dramatic increase in the use of FODS, particularly among older adults in the USA.

Although widely available, FODS are not subject to approval and oversight by the FDA, as for over-the-counter (OTC) drugs, so their content and chemical integrity are not regulated in a rigorous manner [34]. FODS are not in the same category or regulated as OTC or prescription drugs by the FDA. Additionally, their efficacy and safety are not assessed prior to marketing as they are classified by the FDA as a food product [34]. And the low EPA and DHA content of various FODS may require patients to take 10 or more capsules per day in order to attempt to reach the same therapeutic dose (up to 4 g/day) available in a prescription form of O3FA.

Unlike the purification processes used for prescription products, oils extracted from marine animals through large-scale industrial production are a common source for FODS. The fish oil is often a by-product generated during the isolation of protein for animal feed [35, 36]. Due to their chemical structure that includes multiple unsaturated double bonds, O3FAs are highly vulnerable to damage from oxygen free radicals during such extraction procedures. For example, the harvested fish are subjected to 100 °C temperatures in order to isolate the protein components [36]. As a result, O3FAs undergo significant oxidative modification in a manner that is only accelerated in the presence of light and contaminants [36]. Free radical modification of O3FAs would be expected to negate their biologic activity and interfere with any potential clinical benefits associated with the dietary use of FODS [37, 38].

Independent studies from various laboratories have verified concerns about the O3FA content and chemical integrity of FODS. A study funded by the U.S. Department of Agriculture and published in 2015 reported that, of 47 FODS examined, only ten had EPA levels at or above that indicated on their labels, while only 12 had reported amounts of DHA; 74% of the supplements contained less than the stated label amounts of EPA or DHA [39]. In a similar study conducted in New Zealand, 32 FODS were analyzed for fatty acid content of which only 9% had O3FA levels consistent with stated label amounts [40]. In addition, more than 80% of the supplements were found to have unacceptably high levels of lipid peroxides, an indication of lipid decomposition. Of the products tested, only three (8%) met international standards for acceptable peroxide and total oxidation levels [40]. FODS sold in North America have also been shown to have unacceptably high levels of lipid peroxides [41]. Such elevated peroxide values compromise the biological benefits of O3FAs as recently reported [42•].

The fatty acid content of leading FODS (by sales) in the USA was recently analyzed with respect to EPA, DHA, and other oils such as saturated fats [42•]. The extent of oxidative damage of the oils in these FODS was also measured and compared to those in an FDA-approved prescription product [42•]. The results of this analysis showed that more than 30 fatty acids were identified in these popular FODS, including as many as ten to 14 different saturated fatty acids, comprising more than a third of the total fatty acid content. Additionally, O3FA levels varied widely among the FODS, including levels of EPA and DHA by several fold. This study also measured primary and secondary products of oxidation associated with fatty acids containing multiple double bonds, such as O3FAs [42•]. All of the widely available FODS exceeded recommended maxima for these oxidation products. By contrast, no significant levels of oxidation products or other unfavorable oils such as saturated fat were found in the O3FA prescription product.

The biological activity of the O3FAs isolated from a leading FODS was compared to non-oxidized and oxidized preparations of EPA and DHA to determine their effects on atherogenic small dense LDL-C (sdLDL) oxidation [42•]. Oxidation of sdLDL was inhibited by more than 95% (*p* < 0.001) when treated with non-oxidized O3FAs but was not inhibited by oxidized O3FAs or the FODS isolate, which contained both oxidized and non-oxidized O3FAs. The clinical translation of the lack of biological effect from oxidized FODS has been reported to include negative therapeutic effects on blood lipid levels [37] and a lack of intended effectiveness on lipid or inflammatory parameter levels [43].

## Distinct Roles of EPA and DHA in Cellular Function and Atherosclerosis

**Fig. 1:**
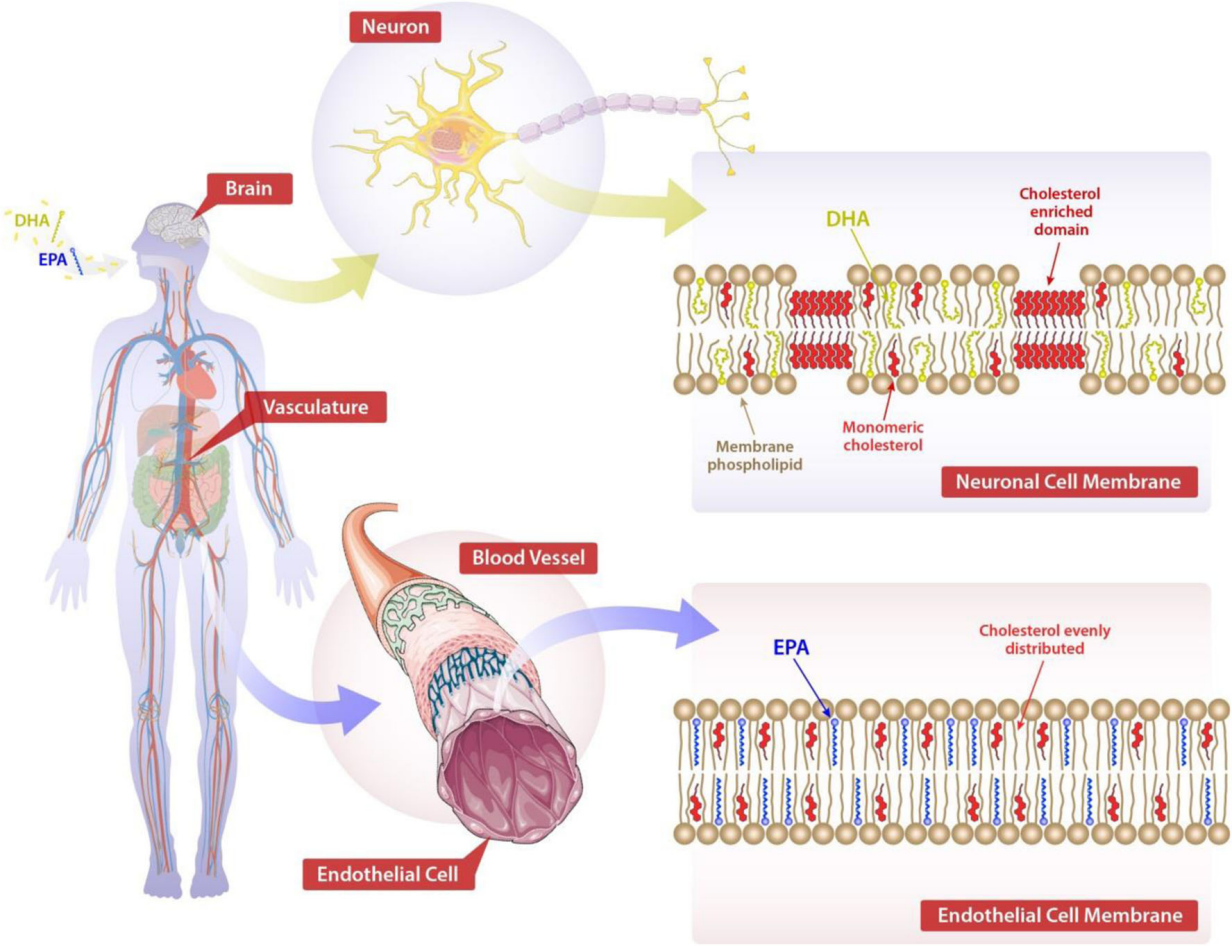
Hypothesized effects of EPA and DHA on endothelial and neuronal cell membrane structural organization, respectively, based on model membrane experiments. DHA is proposed to undergo rapid conformational changes in the neuronal cell plasma membrane where it may promote the formation of cholesterol-rich lipid domains and fluidity—a structural feature shown to be essential to neuronal function. EPA, by contrast, is proposed to intercalate into the membrane phospholipid hydrocarbon core region where it inhibits free radical propagation while preserving a more homogenous cholesterol distribution [13••, 14, 43, 44, 47, 48•, 49•]. Note: This figure contains graphic elements that were modified from Servier Medical Art (http://smart.servier.com/), licensed under a Creative Common Attribution 3.0 Generic License

O3FAs play an essential role in the structure and function of cellular membranes in various tissues throughout the human body. These molecules influence membrane organization, including lipid raft formation, as well as membrane fluidity. O3FAs are metabolized for energy and serve as precursors to important lipid mediators that influence inflammation. These bioactive lipids include eicosanoids, prostaglandins, leukotrienes, and resolvins [44]. Recent research has demonstrated that EPA and DHA have distinct tissue distributions where they influence target organs in different ways (Fig. 1). EPA has been shown to associate with atherosclerotic plaque membranes in blood vessels where it interferes with lipid oxidation and various signal transduction pathways linked to inflammation and endothelial dysfunction [45] as reviewed in Table 2. The basis for these benefits may be, in part, the result of direct effects of EPA on plaque development and stability [17•]. In particular, its lipophilic structure and molecular space dimensions allow EPA to insert efficiently into lipoprotein particles and cell lipid membranes where it scavenges free radicals. In contrast to EPA, DHA serves essential functions in nervous tissues where it is abundant and has pronounced effects on neuronal and retinal membrane organization [46, 47].

**Table 2:** Effects of EPA on plaque progression [2•, 45]

Under conditions of…	EPA increases…	EPA decreases…
Endothelial dysfunction and oxidative stress	• Endothelial function • NO bioavailability	• Cholesterol crystalline domains • oxLDL • RLP-C • Adhesion of monocytes • Macrophages • Foam cells
Inflammation and plaque growth	• EPA/AA ratio • IL-10	• IL-6 • ICAM-1 • hsCRP • Lp-PLA_2_
Unstable plaque	• Fibrous cap thickness • Lumen diameter • Plaque stability	• Plaque volume • Arterial stiffness • Plaque vulnerability • Thrombosis • Platelet activation

*AA* arachidonic acid, *EPA* eicosapentaenoic acid, *hsCRP* high-sensitivity C-reactive protein, *ICAM-1* intercellular adhesion molecule 1, *IL-6* interleukin 6, *IL-10* interleukin 10, *Lp-PLA*_*2*_ lipoprotein-associated phospholipase A, *MMPs* matrix metalloproteinases, *oxLDL* oxidized low-density lipoprotein, *RLP-C* remnant lipoprotein cholesterol

Several lines of evidence show that EPA and DHA differ in their antioxidant properties as well as in their apparent effects on membrane lipid structure and dynamics. The antioxidant effects of EPA are attributed to its ability to quench reactive oxygen species associated with cellular membranes and lipoproteins. Following intercalation into the lipid particle or membrane, the multiple double bonds associated with EPA facilitate electron stabilization mechanisms that inhibit free radical propagation. The antioxidant effects of EPA could not be reproduced with vitamin E or other FDA-approved, TG-lowering agents, under normal or hyperglycemic conditions in vitro [13••, 48•, 49•]. We also observed that the antioxidant activity of EPA could not be reproduced over time with DHA in lipoprotein particles [48•].

To elucidate the basis for these differences in antioxidant function, small angle x-ray diffraction approaches were used to demonstrate that EPA occupies a distinct area in the membrane as compared with DHA [50•]. EPA increased membrane hydrocarbon core electron density over a broad area, indicating an energetically favorable and extended orientation for EPA. By contrast, DHA interacted with the phospholipid head group region with coincident decreases in the hydrocarbon core electron density, a confirmation of its increased molecular volume or disorder. These differences in membrane distribution are attributed to the additional carbon atoms and double bond of DHA, which produces rapid molecular changes that lead to increased lipid disorder that correspond to limitations in its antioxidant capacity [13••, 14, 15, 48•, 51].

The interaction between O3FAs and cholesterol is of particular importance because cholesterol content and organization have profound effects on the overall structure and function of the cell membrane. DHA, for example, has been shown to isomerize through each of its possible confirmations within 50 ns after being added to biological membranes [52]. High acyl chain flexibility and rapid conformational changes are thought to interfere with the close association of O3FAs with cholesterol molecules, which have a rigid steroid ring structure and are less flexible in their membrane disposition [53]. By contrast, EPA does not undergo the same rapid conformational changes as DHA, allowing it to freely distribute with cholesterol and other lipids throughout the membrane bilayer. As a result of this differential lipid interaction, DHA has been observed to promote cholesterol-rich domains in model membranes while EPA has no such effect [13••]. In dietary and cellular models of atherosclerosis, cholesterol has been shown to accumulate and form distinct domains in cellular membranes [54, 55]. These domains are believed to precipitate the formation of toxic extracellular crystals that induce cell apoptosis and necrosis, hallmark features of the unstable atherosclerotic plaque [56–60]. Along with oxidized LDL, cholesterol crystals are also a primary activator of nucleotide-binding domain, leucine-rich-containing family, pyrin domain-containing-3 (NLRP3) inflammasomes, which regulate caspase-1 and its associated processing of pro-interleukin 1 beta (IL-1β) into an active cytokine that initiates inflammation in atherosclerosis [61].

We have also observed that the antioxidant effects of EPA in model membranes and various ApoB particles could not be reproduced by other TG-lowering agents such as niacin, gemfibrozil, and fenofibrate [48•, 49•]. The antioxidant effects of EPA in highly atherogenic LDL-C subfractions such as sdLDL from human subjects were actually enhanced in combination with atorvastatin under in vitro conditions [48•]. This unexpected finding indicates a shared location for these two amphipathic molecules where their intermolecular interactions further stabilize unpaired lipid free radicals and thereby reduce oxidative damage. Thus protected, the non-oxidized LDL-C particle would be less atherogenic and more efficiently cleared from the circulation.

In patients with coronary artery disease (CAD), treatment with EPA has also been shown to improve HDL function. Specifically, HDL isolated from these patients showed enhanced cholesterol efflux and improved HDL activities, including antioxidant and anti-inflammatory effects [62]. In a recent study using isolated human endothelial cells, EPA-enriched HDL inhibited cytokine-stimulated vascular cell adhesion molecule 1 (VCAM-1) expression and increased resolvin E3 production [63•]. HDL treatment also enhanced cholesterol efflux following EPA incorporation [63•]. The lipophilic structure and molecular space dimensions of EPA allow it to insert more efficiently into the HDL particle, with improved antioxidant function, as compared to DHA [64•].

Thus, EPA has direct vascular effects that have been well characterized in cellular and animal models of atherosclerosis and corroborated by various clinical investigations. These studies show that EPA treatment is associated with reduced inflammation and improved plaque stabilization [45]. The distinct location of EPA in the membrane and lipoprotein particles may explain certain differences in its vascular effects when compared to other TG-lowering agents and even DHA. EPA intercalates into the membrane with its long axis parallel to the phospholipid acyl chains, potentially allowing EPA to concentrate efficiently in endothelial and other membranes associated with atherosclerotic plaque. These findings support a mechanistic basis for a potential benefit with EPA in reducing cardiovascular risk as is being currently tested in ongoing clinical trials.

## Effects of Omega-3 Fatty Acids and Statins on Endothelial Function

Endothelial cell (EC) dysfunction is causally related to atherosclerosis and is associated with increased cardiovascular risk [65–67]. As such, treatments leading to the reversal of EC dysfunction may lead to benefits in CAD. While both EPA and statins have been shown separately to improve EC function, their effects in combination have only recently been evaluated in experimental models [68•]. EPA can reverse parameters of endothelial dysfunction caused by exposure to oxLDL or high glucose. Quantitative differences suggest that this effect is enhanced in the presence of atorvastatin active metabolite (ATM). Combination treatment with EPA and ATM has also demonstrated preventative benefit in isolated human EC. This endothelial function improvement was specifically evidenced by favorable increases in the EC ratio of nitric oxide (NO) to peroxynitrite (ONOO^−^) release. In these cells, EPA generated additional benefit with respect to endothelial function as compared to other TG-lowering agents when combined with ATM [68•].

EPA-mediated effects on the endothelial NO/ONOO^−^ release ratio were independent of any changes in endothelial nitric oxide synthase (eNOS) expression, suggesting that the mechanism responsible for this benefit is related to eNOS efficiency rather than an increase in the total amount of enzyme [68•]. This suggests an improvement in eNOS coupling. When eNOS uncoupling occurs, excess O_2_^−^ is generated instead of NO, which decreases NO bioavailability and increases LDL-C oxidation. Increased LDL, and more specifically oxLDL, reduces the EC NO/ONOO^−^ release ratio, demonstrating that dyslipidemia may be causally related to endothelial dysfunction as a result of eNOS uncoupling, a process which may be inhibited with EPA and ATM. Pretreatment of human ECs with EPA and ATM prior to oxLDL exposure also demonstrated a favorable effect on endothelial function. Thus, interactions between EPA and ATM may be related to their similar distributions in the lipid environment of cell membranes and lipid particles, as well as their shared antioxidant properties [49•].

Oxidized LDL contributes to endothelial dysfunction, vascular inflammation, and other processes involved in the development of atherosclerosis. Several lines of evidence suggest that sdLDL is highly atherogenic as compared to larger LDL particles [69–73], especially since sdLDL is more susceptible to oxidative modification as compared to LDL [74, 75]. EPA-mediated protection of ApoB-containing particles, particularly LDL and sdLDL, has been demonstrated in various experimental models [48•]. EPA inhibits oxidation in ApoB-containing particles for a longer period of time than DHA, suggesting that EPA may have more sustained antioxidant benefits than DHA. Oxidized lipids associated with lipoprotein particles contribute to vascular inflammation [76–78]. Evidence shows that elevated sdLDL, in particular, leads to a higher risk of CAD [69, 73]. These findings suggest that the effects of EPA on sdLDL levels and other ApoB containing particles could be clinically relevant given the atherogenicity associated with their oxidation.

The effects of EPA and ATM on endothelial cells in vitro were extended to an ex vivo model using a rodent model [68•]. While both EPA and ATM showed separate benefits, combination treatment exhibited additional improvement regarding NO bioavailability in rat tissues under conditions of either hyperglycemia alone or with simultaneous exposure to oxLDL [68•]. These data suggest that treatments, such as EPA and ATM, that improve NO bioavailability may have broader therapeutic effects in CAD prevention. The endothelial benefits observed ex vivo may in part help to explain reduced CV events observed for hypercholesterolemic patients that received EPA in addition to statin treatment [11]. EPA may have direct effects on endothelial function as compared to other TG-lowering agents, which have failed to reduce CV events as compared to statin treatment alone [6–9].

## Effects of Omega-3 Fatty Acids on Cholesterol Crystal Formation

Cholesterol is an abundant neutral lipid present in the plasma membrane of mammalian cells in amounts ranging from 30 to 50% of total membrane lipids. It has a fundamental role in membranes by modulating lipid bilayer structure and dynamics. It is also involved in cell signaling pathways and serves as precursor of various hormones, bile acids, and vitamin D. Atherosclerosis is characterized by excessive accumulation of cholesterol and oxidative damage in endothelial cells, smooth muscle cells, and eventually macrophages in the arterial cell wall. Oxidative damage and excessive cholesterol accumulation induce the formation of distinct immiscible cholesterol crystalline domains in cellular membranes [55, 79]. Over time, these cholesterol domains can precipitate the formation of insoluble, extracellular cholesterol crystals, a hallmark feature of the atherosclerotic plaque [80]. Cholesterol crystals have been observed using microscopy approaches and are evidenced by sharp, jagged edges [58] that can destabilize the fibrous cap of the plaque [57], leading to local inflammation and thrombus formation. The association of cholesterol crystals with plaque has been reported in patients that have experienced myocardial infarction [56]. These domains are not formed exclusively from excess cholesterol. Membranes exposed to oxidative stress or high glucose can also induce cholesterol domain formation despite normal cholesterol levels [81]. These crystals are also a primary activator of NLRP3 inflammasomes [61].

Due to its potent antioxidant activity and lipophilicity, EPA significantly inhibited glucose-induced cholesterol crystalline domain formation in model membrane lipid vesicles at pharmacologically relevant concentrations [49•, 82]. The inhibition of cholesterol domain formation by EPA could not be reproduced with other TG-lowering agents or vitamin E [49•]. These findings suggest that EPA preferentially intercalates into the hydrocarbon core of the membrane bilayer where it can trap free radicals with its multiple double bonds. The lack of benefit for vitamin E under these conditions is attributed to its limited lipophilicity and free radical scavenging activity.

Under conditions of high membrane cholesterol levels, EPA was also able to preserve normal membrane structure and lipid organization as compared to DHA or other TG-lowering agents [13••]. Conformational differences between EPA and DHA in the membrane are influenced by surrounding phospholipid and cholesterol molecules. Previous studies have demonstrated that DHA is able to change conformations quickly (on a nanosecond time scale) in the membrane lipid bilayer environment [83], but EPA has a more stable membrane conformation [50•]. Such rapid conformational changes reduce DHA interactions with cholesterol molecules which have a rigid steroid ring structure and its long axis parallel to surrounding phospholipid acyl chains [84, 85]. Thus, DHA actually promotes cholesterol enriched domains as compared to EPA.

## Conclusions

Elevated TGs are an important contributor to residual cardiovascular risk due to their direct effects on plaque formation. Clinical and basic research data indicate that EPA has a beneficial role as an add-on to statin therapy in slowing the development and progression of atherosclerotic disease. EPA and DHA have distinct effects on membrane structure, lipid dynamics, and rates of membrane lipid oxidation. Compared to other TG-lowering therapies, EPA has been found to reduce markers of inflammation, cholesterol crystal formation, endothelial dysfunction, and oxidative modification of various ApoB-containing lipoprotein particles as well as increasing the functionality of HDL. Certain benefits with EPA are markedly enhanced in combination with a statin. The recent results of a large randomized clinical trial have now confirmed the benefit of an EPA-only pharmacologic dose (4 g/day) in statin-treated patients with established cardiovascular disease or diabetes and additional CV risk factors in reducing CV events, including CV mortality. Ongoing mechanistic trials will provide additional insights as to the role of O3FAs in reducing residual cardiovascular risk and progression of coronary atherosclerosis in subjects with well-managed LDL-C but elevated TG levels [29, 86].
